# New perspectives on treatment strategies for patient with acute myeloid leukemia and complex karyotype abnormalities after percutaneous coronary intervention: A case report

**DOI:** 10.1097/MD.0000000000016586

**Published:** 2019-07-26

**Authors:** Jie Wang, Rong Chen, Xiaoqi Lin, Yubo Wang, Jian-Hua Wang, Yun Wu

**Affiliations:** a Department of Pharmacy; b Department of Hematology; c Department of General Medicine, First Affiliated Hospital of Xinjiang Medical University, Urumqi, People's Republic of China.

**Keywords:** acute myeloid leukemia, percutaneous coronary intervention, pharmaceutical care

## Abstract

**Rationale:**

Acute myeloid leukemia (AML), in patients with coronary heart disease (CHD) and treated percutaneous coronary intervention (PCI), is rarely seen in clinic. There are few similar cases reported, and there are no evidence-based medicine guidelines for the treatment.

**Patient concerns:**

A 52-year-old man was diagnosed with coronary atherosclerotic heart disease in November 2011, and received a stent placement in the left anterior descending coronary artery 1 year later. One day after the surgery, his laboratory tests showed pancytopenia.

**Diagnoses:**

Based on precise diagnosis of leukemia, namely cell morphology, immunology, cytogenetics, and molecular biological typing, the patient was diagnosed with AML-M2.

**Interventions:**

The patient received idarubicin with cytarabine in 1st cycles, and single cytarabine regimen was used in 2nd and 3rd cycles for the accumulative toxicity of idarubicin in postinduction chemotherapy. Meanwhile, staged-treatment strategy was implemented by using antiplatelet drugs during different chemotherapy phases, and personalized pharmaceutical care on the basis of the recognition of potential adverse effects of chemotherapy regimen.

**Outcomes:**

Until now, the disease-free survival in the patient has been over 6 years, and he is still followed up in clinic.

**Lessons:**

Although leukemia accompanied with coronary heart disease, even after receiving the coronary stenting therapy is rarely seen in clinic, the treatment with antiplatelet drugs for post chemotherapy patients with coronary disease is necessary. Clinical pharmacists are supposed to be more proficient in developing personalized drug treatment strategies, especially maintaining the balance between the effect and the risk in difficult and complex cases.

## 1 Introduction

Acute myeloid leukemia (AML) is a hematopoietic malignancy which is characterized by the proliferation of blast cells and the hematopoietic dysfunction of normal bone marrow, and it is the most common type of acute leukemia, with a high mortality rate, among adults in clinic .^[1]^ Patients suffering from AML are more vulnerable to thrombocytopenia due to the inhibited function of normal bone marrow after chemotherapy. For that reason, it is usually required that patients need to get inpatient care during the treatment period to prevent the occurrence of bleeding and other complications, which are both the roots of high mortality.^[2]^

Percutaneous coronary intervention (PCI) is an effective treatment for coronary heart disease (CHD). Acute stent thrombosis is one of the serious complications, it needs dual antiplatelet therapy (DAPT) to prevent abrupt thrombotic vessel closure after PCI.^[3,4]^ Although DAPT, as a secondary prevention, is associated with lower risk of death originating from atherosclerosis progression,^[5]^ recent research results have shown a higher risk of bleeding in patients received DAPT, which can sometimes increase the noncardiovascular mortality.^[6,7]^ However, DAPT, as a secondary prevention, remains questionable, especially when it comes to the patient with AML and CHD together, because it is hard to balance the contradiction between bleeding and thrombosis risk, let alone this kind of case reports are insufficient. Our case report describes a patient with AML after PCI, and we carefully designed pharmaceutical care to help patient get a better treatment.

## 2 Case presentation

**Figure 1:**
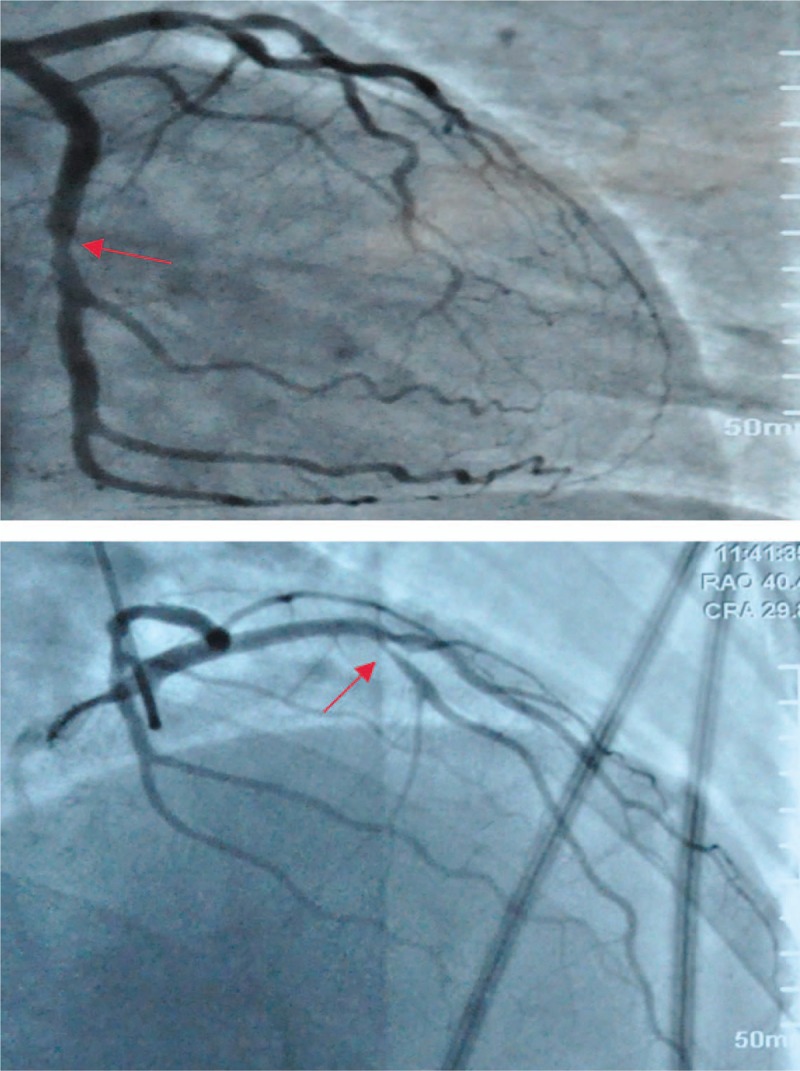
Initial coronary angiogram showed that left anterior descending coronary artery (LAD) and left circumflex branch (LCX) has mild stenosis. In November 2011, initial coronary angiography examination showed that both the left anterior descending coronary artery (LAD) and left circumflex branch (LCX) had 30% to 40% stenosis in the middle portion.

A 52-year-old man was admitted to the hospital on emergency basis due to symptoms of chest tightness for 5 years and palpitation aggravated for 5 days. The patient reported a history of chest tightness, shortness of a breath, and occasional headache persist for 5 years after fast walking, climbing stairs, fatigue, anger, and the symptoms could be alleviated 1 or 2 minutes later. In November 2011, he came to the hospital for a recurrent and unknown chest tightness. Coronary angiography examination observed that both left anterior descending coronary artery (LAD) and left circumflex branch (LCX) morphed into mild stenosis (Fig. 1). The coronary atherosclerotic heart disease was diagnosed, and no change of blood was found in the routine exam (white blood cell count, 9.1 × 10^9^/L; hemoglobin, 165 g/L; and platelets, 272/ × 10^9^/L). After discharging from the hospital, the doctor prescribed him aspirin (100 mg QD), clopidogrel (75 mg QD), atorvastatin (20 mg QD), and metoprolol (12.5 mg BID) for secondary prevention of CHD, but he did not take the medicine as directed.

**Figure 2:**
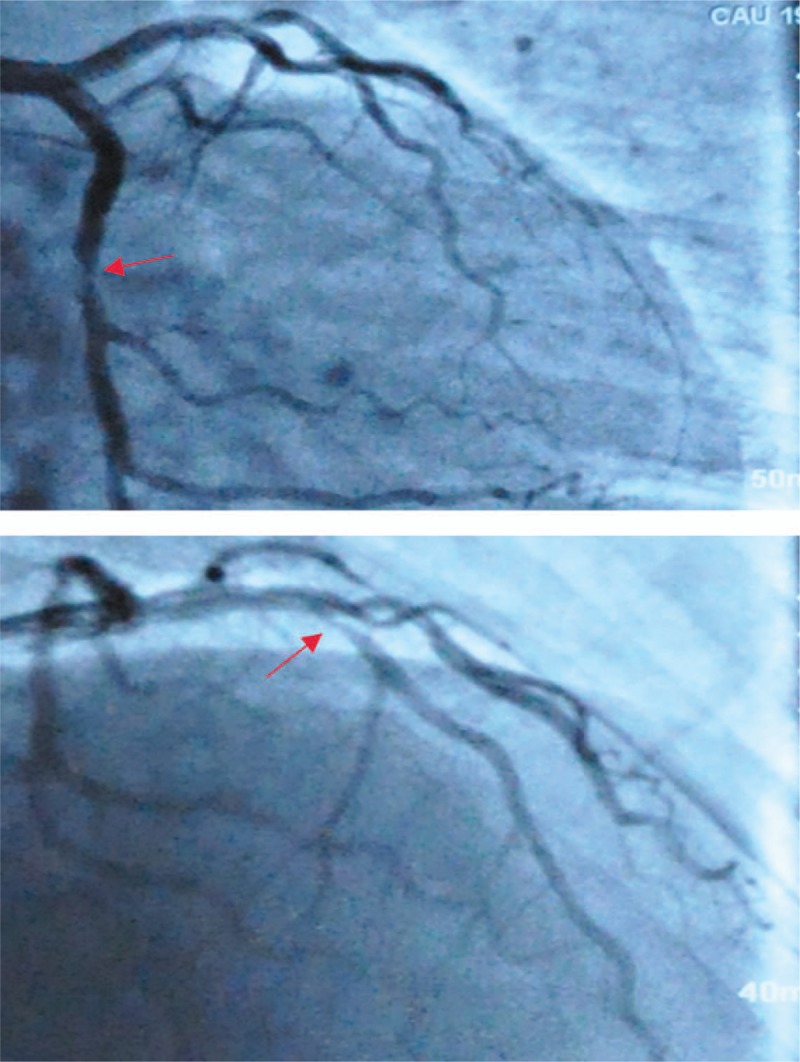
A year later, the coronary angiography examination showed stenosis degree aggravating than earlier. In November 2012, because of chest pain and the other symptoms significantly worse than before, so the patient underwent coronary angiography examination again, the results showed that the 75% localized stenosis was seen in the middle proption of the LAD, and 60% localized stenosis was seen in the middle proption of the LCX.

**Figure 3:**
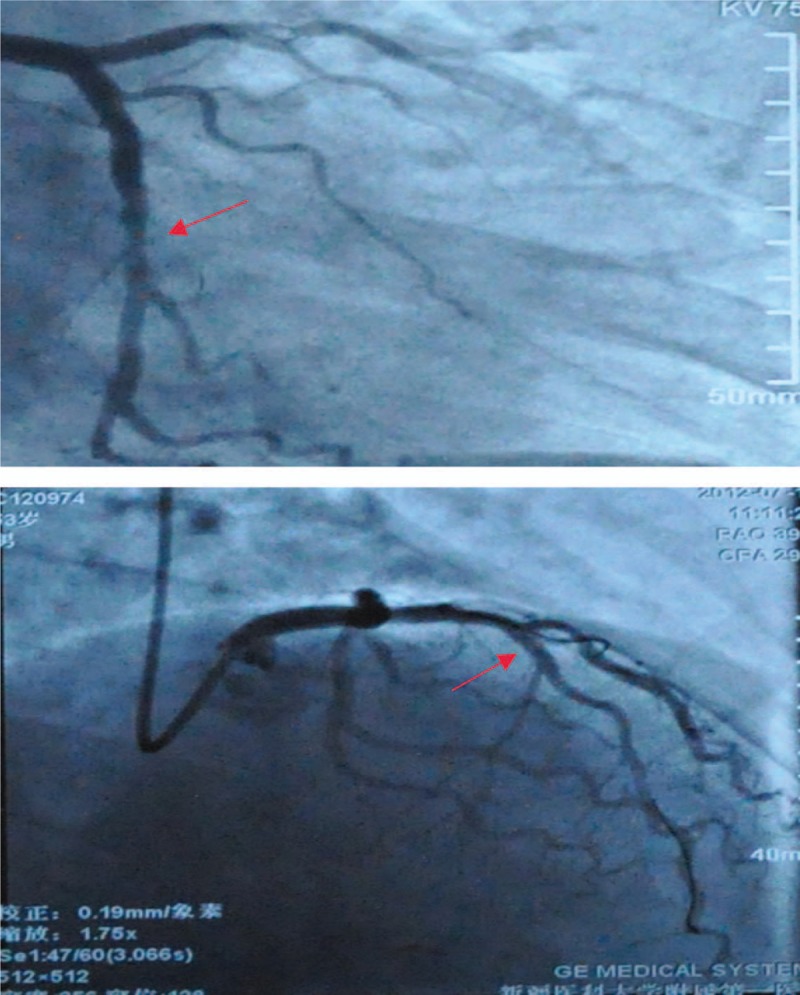
The final coronary angiogram showed a successfully implanted stent at the LAD. The pathological changes were quickly resolved by percutaneous transluminal coronary angioplasty and placement of 1 drug-eluting stents in the LAD.

In July 2012, the patient came back to the emergency department because of chest pain and the other symptoms significantly worsened than ever. Physical examination of normal vital signs revealed there was no obvious petechiae or blood spots found on his skin. Then the patient underwent coronary angiography examination, and the result showed an aggravation of stenosis (Fig. 2), so did percutaneous transluminal coronary angioplasty and placement of 1 drug-eluting stents in the LAD (Fig. 3). His chest pain and pathological changes quickly resolved by PCI procedure, and on a regimen of aspirin (100 mg QD), clopidogrel (75 mg QD).

**Figure 4:**
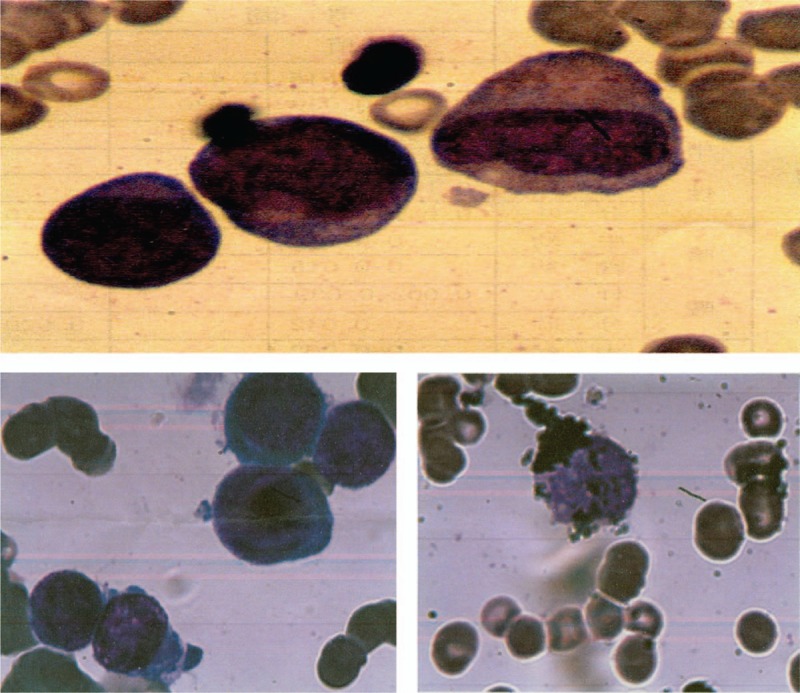
The results of bone marrow examination when the patient had unknown pancytopenia and suspected with hematological diseases. Bone marrow aspiration revealed abundant, immature myeloid cells with coalescent granules, and histochemical staining showed peroxidase staining positive rate approximate 83% and alkaline phosphatase staining rate approximate 2%.

**Figure 5:**
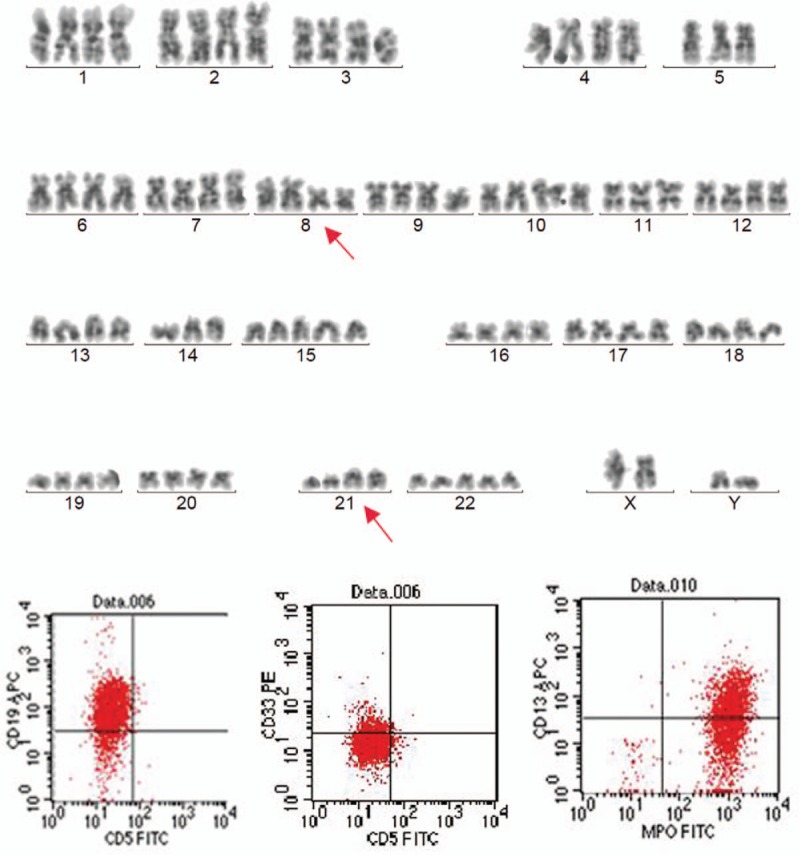
The results of chromosome karyotype analysis and immunophenotype. Conventional cytogenetic analysis was carried out on direct preparations, and immunophenotype was performed on isolated bone marrow mononuclear cells by flow cytometry after collection according to standard procedure. Cytogenetic karyotypes revealed 88∼9,XX,YY,2 × t(8;21)(q22;q22)[20], and immunophenotype suggested myeloid expression with HLA-DR, CD13, CD15, CD19, CD33, CD34, CD38, CD56, CD64, CD117, CD123, MPO.

**Figure 6:**
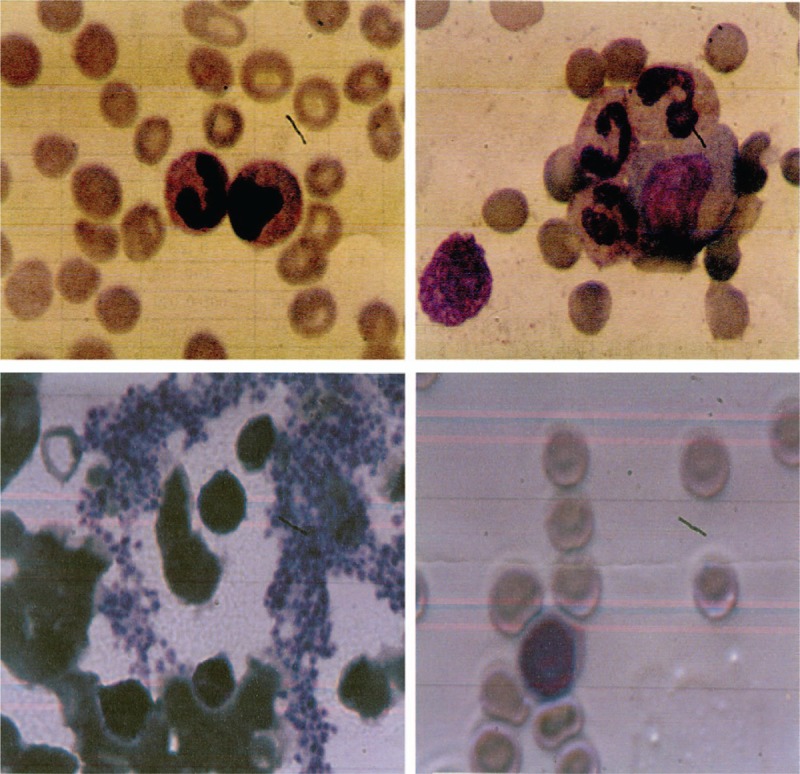
The results of bone marrow and peripheral examination after chemotherapy. Bone marrow morphology that revealed no clusters or collections of blast cells, and peripheral blood smear could not observe bone marrow blast cell.

**Figure 7:**
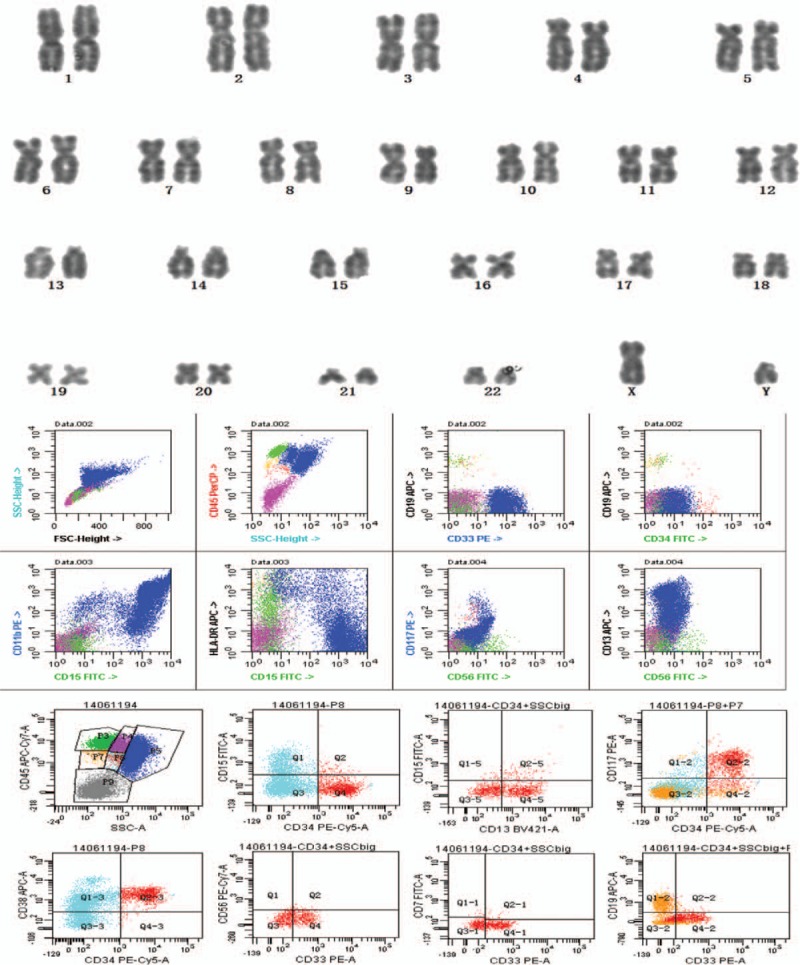
The results of cytogenetic karyotypes and minimal residual disease (MRD) during the patient followed up in clinic. Cytogenetic karyotypes and MRD was detected during the patient followed up in clinic, and the sesults showed continuous complete remission after chemotherapy.

One day later, he was found to have unknown pancytopenia (hemoglobin, 6.9 mg/dL; white blood cell count, 3.4 × 10^9^/L; and platelets, 12 × 10^9^/L), for which no explanation was found and suspected to have hematological diseases. A bone marrow aspirate and biopsy were performed to evaluate thrombocytopenia and it showed the abundance of immature myeloid cells with coalescent granules. Histochemical staining showed the myeloid cells stained positive with myeloperoxidase, so the patient was diagnosed with AML, and the AML was identified as AML-M2 (World Health Organization classification M2; Fig. 4). Cytogenetic karyotypes revealed 88 to 91,XX,YY,2 × t(8;21)(q22;q22)[20], immunophenotype suggested myeloid expression with HLA-DR, CD13, CD15, CD19, CD33, CD34, CD38, CD56, CD64, CD117, CD123, MPO (Fig. 5). Notably, PML/RARa fusion genes were no expression, but ETO fusion genes were expressed as 4.59 × 10^4^. Besides, the mutation testing of C-KIT, FLT-3, and NPM1 was negative. The patient received 7 cycles of IA (idarubicin 10 mg D1-3, cytarabine 200 mg D1-7) and HD-Ara-C (high dose cytarabine, 2000 mg D1,3,5) scheme chemotherapy, and he was in complete remission after chemotherapy (Figs. 6 and 7). To prevent stent thrombosis, the patient received clopidogrel (75 mg QD) monotherapy when the platelet counts between 60 and 100 × 10^9^/L, and combined aspirin (100 mg QD) when the platelet counts greater than 100 × 10^9^/L, wherever stopped all the antiplatelets when the platelet counts less than 60 × 10^9^/L. The disease-free survival in the patient has been over 6 years, and the patient is still being followed up in clinic.

## 3 Discussion

Judging from poor prognostic factors, the patient with AML needed treatment immediately.^[8]^ For patients after PCIs, both conventional anticoagulant and antithrombotic agents are required.^[9]^ But for AML in patients with CHD, few similar cases are reported and no official guidelines and advice exist, making the treatment decision a very difficult and confused clinical issue.^[10]^ We reviewed the reports related to both CHD and AML. It was found that patients with acute promyelocytic leukemia have been reported in many literatures.^[11,12]^ On the one hand, APL is the underlying cause of CHD, and APL-associated thrombosis events are associated with leukemia and the interaction between malignant promyelocytes and host cells.^[13]^ On the other hand, APL patients with bleeding are well known, high incidence of early hemorrhagic deaths remains the greatest contributor to chemotherapy failure.^[14]^ More importantly, bleeding risk elevates after leukemia chemotherapy and postoperative rehabilitation, making how to handle thrombosis in AML more complicated.

Fortunately, the patient had a good prognosis, and prior to making the therapeutic strategies, there are some key issues to be determined in this case, including the adjustment of appropriate regimen selection for antithrombotic therapy based on the periodic change of platelet status, the paradox of bleeding and thrombosis events, and the maintenance of the balance between anthracycline cardiotoxicity and weak cardio function.

### 3.1 The consideration and advice on chemotherapy regimen adjustment

A low platelet in AML makes the therapeutic treatment plan hard to be settled, because it is predictable that platelet count would be decreased during chemotherapy. Moreover, the platelets would decrease further due to myelosuppression. Therefore, supportive therapies, like blood transfusion, need to be employed, if necessary, to lower the risk for bleeding.^[15]^ Meanwhile, risks of bleeding and thrombosis are both elevated in leukemia, and PCI can also raise the risk in thrombosis, so the balance among several aspects is of great significance.^[10,16]^ Thus, an integrated therapeutic regimen should be carefully designed in the early phase treatment for this patient, and supportive care should be commenced at once the completion of chemotherapy to avoid a severer level of myelosuppression.

Considering the risk in bleeding is high prior to having chemotherapy, we decided to stop using Low-Molecular-Weight Heparins Sodium and Aspirin immediately. Considering thrombocytopenia and prophylaxis of stent thrombosis, clopidogrel should be still prescribed combined (75 mg QD) with individual anticoagulant and anti-thrombosis therapy.^[17]^

Furthermore, according to the clinical guideline, idarubicin (10 mg, IVGTT, D1-3) and cytarabine (200 mg, IVGTT, D1-7) were chosen as primary induction chemotherapy regimen.^[15]^ Then single cytarabine regimen (2000 mg, IVGTT, D1, 3, 5) was used in 2nd and 3rd cycles every 3 cycles to reduce the accumulative toxicity of idarubicin in postinduction chemotherapy.^[18]^ There was an attenuation in the result of bone marrow biopsy test after the induction chemotherapy. The cardiac function was monitored during therapy time, and there was no related adverse event occurred.

In addition, chemotherapy-induced symptoms need to be controlled and relieved clinically by hydration, urine alkalized, etc. Cytarabine has renal toxicity and neurotoxicity and patients receiving high-dose cytarabine therapy are at high risk of cerebellar toxicity monitored by neurologic assessment, including tests for nystagmus, slurred speech, and dysmetria that should be performed before each dose of cytarabine.^[19]^ Additionally, the accumulative dose of anthracycline worth attention, the protection agent Dexrazoxane should be administrated before 30 minutes of idarubicin.^[20]^

### 3.2 The staged-treatment strategy for anticoagulation therapy

It is well known that aspirin has an irreversible effect on thromboxane A2 (TXA_2_) from pharmacological perspective. Platelet aggregation triggers the reduction of TXA_2_, and during the long term of treatment, it could maintain a constant level in plasma.^[21]^ This would make its synergistic effect, with Clopidogrel, on inhibiting the platelet aggregation promoted. Thus, the combination of these 2 drugs could increase the risks of bleeding.^[22]^ Moreover, the metabolism of Clopidogrel relies on the CYP2C19, and the genotype of CYP2C19 ×1×1 of patients, which means the concentration of the active metabolite of Clopidogrel could be high, so that is the reason why single Clopidogrel could have a satisfying effect of antithrombosis in this case.^[23,24]^

Therefore, the therapeutic strategy for the patient in this case was designed according to following steps. Low-Molecular-Weight Heparins Sodium and Aspirin was stopped immediately due to thrombocytopenia and prophylaxis of stent thrombosis. Because of a high coagulation state resulting from the release of the procoagulant from leukemia cell, Clopidogrel (75 mg QD) remained to be prescribed with individualized antithrombosis therapy. It was concluded that Clopidogrel needed to be maintained and aspirin ought to be stopped under the condition of platelet counts less than 100 × 10^9^/L. Clopidogrel could be stopped when the platelet counts less than 60 × 10^9^/L in case of the high risks of bleeding. The combination treatment could be restored after chemotherapy for the recovery of blood cell counts. Finally, the suggestion was taken by the physician to make an adjusted regimen. There were no bleeding and thrombosis any more, with coagulation function being normal, during the whole process of treatment.

### 3.3 Other cautions for treatment procedures

The chance of fungal infection increased remarkably because of chemotherapy. The use of azoles combined with idarubicin and cytarabine elevates the toxicity of drugs compared with using alone, and weakens the effect of antifungi agents.^[25]^ Consequently, the selection of antifungi agents should be cautioned.

Statins, as a regular preventive medication for atherosclerosis, would cause hepatic dysfunction and affect the coagulation.^[26]^ Pharmacists give a suggestion that the functions of liver and coagulation are needed to be monitored to avoid bleeding. Even though the metabolisms of Atorvastatin and Clopidogrel are both determined by CYP 3A4, the metabolic capability of CYP 3A4 is not saturated for the combination of these 2 drugs with a regular dose, and furthermore, there is an increase in the activity of nitric oxide synthetase in the platelet to promote the disaggregation of platelet.^[27]^ Therefore, statins play an essential role in the atherosclerotic ischemia therapy.

Currently, based on the evidence, there is a correlation between stent thrombosis and special high coagulation state of the mutation on factor V and antiphospholipid syndrome.^[28]^ High coagulation state could be induced by certain solid tumor particularly in some adenocarcinoma, and it is reported that the incidence of AML shows a correlation with hyperhomocysteinemia, and noted that the level of homocysteine would enhance the production of thrombus.^[29]^ In summary, the therapeutic experience for cancer combined with thrombus is lacked and there are few similar reports.

According to this case, it is found that, besides the individual genetic factor, other aspects that would influence the treatment, for instance, the size, type and position of the stent; postoperative stent status, complications and, medication tolerance and dose during treatment should be taken into consideration. According to the guidelines, there is insufficient evidence to support PCI for the cancer patients with stable angina pectoris in recent years. It is believed that more similar cases will be reported with an increased incidence of malignancy and growing number of the stent, and more evidence will provide a wiser strategy for the individual treatment. Further proof for the PCI indication of patients with malignancy, antithrombosis and anticoagulation pharmacotherapy will be studied.

### 3.4 Ethical approval

All procedures performed in studies involving human participants were in accordance with the ethical standards of the institutional research committee at which the studies were conducted and with the 1964 Helsinki declaration and its later amendments or comparable.

### 3.5 Patient consent statement

Informed Consent: Patient has provided informed consent for publication of the case. A copy of the consent form is available for review upon request.

## Acknowledgments

The authors thank Zhengyu Song and Kai Wang, PhD (School of Clinical Pharmacy, China Pharmaceutical University, Jiangsu, China), who help us revise the manuscript cordially and critically.

## Author contributions

**Conceptualization:** Jie Wang, Yun Wu.

**Data curation:** Jie Wang, Xiaoqi Lin, Yun Wu.

**Formal analysis:** Yubo Wang.

**Investigation:** Rong Chen, Xiaoqi Lin, Yun Wu.

**Project administration:** Jie Wang.

**Supervision:** Jianhua Wang.

**Validation:** Jianhua Wang.

**Writing – original draft:** Jie Wang, Yun Wu.

**Writing – review & editing:** Yubo Wang.

## References

[1] Short NJ Rytting ME Cortes JE Acute myeloid leukaemia. Lancet 2018;392:593–606. 30078459 10.1016/S0140-6736(18)31041-9 PMC10230947

[2] Ho G Jonas BA Li Q Early mortality and complications in hospitalized adult Californians with acute myeloid leukaemia. Br J Haematol 2017;177:791–9. 28419422 10.1111/bjh.14631 PMC5444943

[3] Mahmud E Ben-Yehuda O Percutaneous coronary intervention in acute coronary syndrome: completing the job saves lives. J Am Coll Cardiol 2018;72:2000–2. 30336822 10.1016/j.jacc.2018.08.2129

[4] Kedhi E Fabris E van der Ent M Six months versus 12 months dual antiplatelet therapy after drug-eluting stent implantation in ST-elevation myocardial infarction (DAPT-STEMI): randomised, multicentre, non-inferiority trial. BMJ 2018;363:k3793. 30279197 10.1136/bmj.k3793 PMC6167608

[5] Fanari Z Malodiya A Weiss SA Long-term use of dual antiplatelet therapy for the secondary prevention of atherothrombotic events: meta-analysis of randomized controlled trials. Cardiovasc Revasc Med 2017;18:10–5. 10.1016/j.carrev.2016.07.006 PMC5250594 27477306

[6] Généreux P Giustino G Witzenbichler B Incidence, predictors, and impact of post-discharge bleeding after percutaneous coronary intervention. J Am Coll Cardiol 2015;66:1036–45. 26314532 10.1016/j.jacc.2015.06.1323

[7] Capodanno D Gargiulo G Buccheri S Meta-analyses of dual antiplatelet therapy following drug-eluting stent implantation: do bleeding and stent thrombosis weigh similar on mortality. J Am Coll Cardiol 2015;66:1639–40. 26429096 10.1016/j.jacc.2015.05.085

[8] Nabhan C Kamat S Karl KJ Acute myeloid leukemia in the elderly: what constitutes treatment value. *Leuk Lymphoma*. 2019;60:1164–1170. 30407103 10.1080/10428194.2018.1520992

[9] Palmerini T Benedetto U Bacchi-Reggiani L Mortality in patients treated with extended duration dual antiplatelet therapy after drug-eluting stent implantation: a pairwise and Bayesian network meta-analysis of randomised trials. Lancet 2015;385:2371–82. 25777667 10.1016/S0140-6736(15)60263-X

[10] Sargsyan Z Higgins C Alexandrescu S Acute promyelocytic leukemia as a cause of intracoronary drug-eluting-stent thrombosis. Tex Heart Inst J 2012;39:416–9. 22719158 PMC3368467

[11] Choudhry A DeLoughery TG Bleeding and thrombosis in acute promyelocytic leukemia. Am J Hematol 2012;87:596–603. 22549696 10.1002/ajh.23158

[12] Özkurt ZN Aypar E Sarifakiogullari S Acute myocardial infarction as a finding of acute promyelocytic leukemia-related coagulation disorder. Blood Coagul Fibrinolysis 2015;26:949–52. 26523810 10.1097/MBC.0000000000000331

[13] Rashidi A Silverberg ML Conkling PR Thrombosis in acute promyelocytic leukemia. Thromb Res 2013;131:281–9. 23266518 10.1016/j.thromres.2012.11.024

[14] Chang H Kuo MC Shih LY Acute promyelocytic leukemia-associated thrombosis. Acta Haematol 2013;130:1–6. 23343825 10.1159/000345833

[15] O’Donnell MR Tallman MS Abboud CN Acute Myeloid Leukemia, Version 3.2017, NCCN Clinical Practice Guidelines in Oncology. J Natl Compr Canc Netw 2017;15:926–57. 28687581 10.6004/jnccn.2017.0116

[16] Staib P Forsch S Niedeggen A [Percutaneous coronary intervention in a patient with acute myeloid leukemia]. Dtsch Med Wochenschr 2012;137:1092–5. 22588653 10.1055/s-0032-1305014

[17] Pavlovic M Apostolovic S Stokanovic D The association between clopidogrel and 2-oxo-clopidogrel plasma levels and the long-term clinical outcome after acute myocardial infarction. Int J Clin Pharmacol Ther 2019;57:82–93. 30431425 10.5414/CP203190

[18] Babiker HM McBride A Newton M Cardiotoxic effects of chemotherapy: a review of both cytotoxic and molecular targeted oncology therapies and their effect on the cardiovascular system. Crit Rev Oncol Hematol 2018;126:186–200. 29759560 10.1016/j.critrevonc.2018.03.014

[19] Andresen V Gjertsen BT Drug repurposing for the treatment of acute myeloid leukemia. Front Med (Lausanne) 2017;4:211. 29238707 10.3389/fmed.2017.00211 PMC5712546

[20] Vachhani P Shin S Baron J Dexrazoxane for cardioprotection in older adults with acute myeloid leukemia. Leuk Res Rep 2017;7:36–9. 28462084 10.1016/j.lrr.2017.04.001 PMC5402627

[21] Kerry L Albert F Antiplatelet therapy in acute coronary syndrome. Eur Cardiol 2017;12:33–7. 30416549 10.15420/ecr.2016:34:2 PMC6206448

[22] Diener HC Bogousslavsky J Brass LM Aspirin and clopidogrel compared with clopidogrel alone after recent ischaemic stroke or transient ischaemic attack in high-risk patients (MATCH): randomised, double-blind, placebo-controlled trial. Lancet 2004;364:331–7. 15276392 10.1016/S0140-6736(04)16721-4

[23] Simon T Verstuyft C Mary-Krause M Genetic determinants of response to clopidogrel and cardiovascular events. N Engl J Med 2009;360:363–75. 19106083 10.1056/NEJMoa0808227

[24] Collet JP Hulot JS Pena A Cytochrome P450 2C19 polymorphism in young patients treated with clopidogrel after myocardial infarction: a cohort study. Lancet 2009;373:309–17. 19108880 10.1016/S0140-6736(08)61845-0

[25] Colburn DE Giles FJ Oladovich D In vitro evaluation of cytochrome P450-mediated drug interactions between cytarabine, idarubicin, itraconazole and caspofungin. Hematology 2004;9:217–21. 15204103 10.1080/10245330410001701585

[26] Ehrenstein MR Jury EC Mauri C Statins for atherosclerosis—as good as it gets. N Engl J Med 2005;352:73–5. 15635116 10.1056/NEJMe048326

[27] Farid NA Small DS Payne CD Effect of atorvastatin on the pharmacokinetics and pharmacodynamics of prasugrel and clopidogrel in healthy subjects. Pharmacotherapy 2008;28:1483–94. 19025429 10.1592/phco.28.12.1483

[28] Eshtehardi P Eslami M Moayed DA Simultaneous subacute coronary drug-eluting stent thrombosis in two different vessels of a patient with factor V Leiden mutation. J Cardiovasc Med (Hagerstown) 2008;9:410–3. 18334899 10.2459/JCM.0b013e3282eee98b

[29] Fursevich D Zuchowski C Limback J Leukemic ischemia: a case of myocardial infarction secondary to leukemic cardiac involvement. Case Rep Cardiol 2017;2017:7298347. 28848680 10.1155/2017/7298347 PMC5564183

